# Prognostic Factors of Survival in Patients with Surgically Treated Penile Squamous Cell Carcinoma: A Retrospective Cohort Analysis

**DOI:** 10.3390/cancers18060952

**Published:** 2026-03-14

**Authors:** Andrei Andreșanu, Constantin Gîngu, Dragoș Eugen Georgescu, Mihaela Roxana Oliță, Mihai Adrian Dobra, Cristian Mirvald, Bogdan Obrișcă, Mihai-Adrian Eftimie, Ioanel Sinescu

**Affiliations:** 1 Department of Urology, Carol Davila University of Medicine and Pharmacy, 020021 Bucharest, Romania; andrei.andresanu@drd.umfcd.ro (A.A.); constantin.gingu@umfcd.ro (C.G.); mihai.dobra@umfcd.ro (M.A.D.); cristian.mirvald@umfcd.ro (C.M.); ioanel.sinescu@umfcd.ro (I.S.); 2 Center of Uronephrology and Kidney Transplantation, Fundeni Clinical Institute, 022328 Bucharest, Romania; bogdan.obrisca@umfcd.ro; 3 Department of Surgery, Carol Davila University of Medicine and Pharmacy, 020021 Bucharest, Romania; mihai.eftimie@umfcd.ro; 4 Department of General Surgery “I. Juvara”, “Dr. I. Cantacuzino” Clinical Hospital, 73206 Bucharest, Romania; 5 Department of Anesthesiology and Intensive Care, Carol Davila University of Medicine and Pharmacy, 020021 Bucharest, Romania; mihaela.olita@umfcd.ro; 6 Anesthesiology and Intensive Care, Fundeni Clinical Institute, 022328 Bucharest, Romania; 7 Department of Nephrology, Carol Davila University of Medicine and Pharmacy, 020021 Bucharest, Romania; 8 Department of Surgery, Fundeni Clinical Institute, 022328 Bucharest, Romania

**Keywords:** penile cancer, squamous cell carcinoma, prognostic factors, survival analysis, urethral invasion, Cox regression, TNM staging, eastern Europe, lymph node metastasis

## Abstract

**Background/Objectives**: Penile squamous cell carcinoma (PSCC) is a rare malignancy with a potential major impact on survival. Prognostic assessment remains challenging, particularly in underrepresented eastern European populations, where region-specific evidence is lacking. This paper aimed to identify independent predictors of overall survival in surgically treated patients with PSCC from a Romanian high-volume tertiary center. **Methods**: This retrospective cohort study analyzed 60 patients who were surgically treated for PSCC between October 2020 and December 2024. Univariate and multivariate Cox proportional hazards regression analyses were performed to identify independent prognostic factors. **Results**: The mean patient age was 62 ± 12 years. T-stage distribution showed 30% pT1, 35% pT2, 31.67% pT3, and 3.33% pT4, with 55% of patients presenting with nodal metastases. Univariate analyses demonstrated significant associations between lymphovascular invasion (*p* < 0.001), perineural invasion (*p* = 0.022), and positive surgical margins (*p* = 0.030) and risk of death. Multivariate analysis identified three independent prognostic factors: absence of histologically documented urethral invasion (HR 0.32; *p* = 0.027), T3–T4 disease (HR 8.26; *p* = 0.005 vs. T1), and N3 stage (HR 3.53; *p* = 0.030 vs. N0–N1). Patients without urethral invasion demonstrated significantly longer median overall survival (63 months vs. 11 months). The final three-variable prognostic model demonstrated good discrimination (C-index 0.78), providing a potential practical risk stratification tool. **Conclusions**: Urethral invasion, advanced T-stage, and N3 disease independently predict poor survival in surgically treated PSCC. The identification of urethral invasion as an independent prognostic factor warrants consideration in clinical practice. This is the first study of a Romanian cohort to provide critical data for risk-adapted treatment strategies in underrepresented eastern European populations.

## Simple Summary

Penile cancer is a rare but serious disease. Predicting which patients will have better or worse outcomes after treatment is difficult, especially in eastern Europe, where evidence is limited. We studied 60 patients who underwent surgery for penile cancer at a Romanian tertiary uro-oncology center to identify which factors predict survival. We found that three features independently predicted worse outcomes: advanced tumor stage, extensive lymph node involvement and urethral invasion. Notably, urethral invasion emerged as a newly recognized predictor that is not currently included in standard staging systems. These findings suggest that a three-factor model for predicting survival can be used to counsel patients more accurately, guide treatment intensity and identify individuals at high risk who may benefit from more aggressive therapy.

## 1. Introduction

Penile squamous cell carcinoma (PSCC) represents a rare malignancy with a heterogeneous incidence worldwide. While in Europe and the USA, the incidence of this pathology is 0.94/100,000 and 0.5, respectively, in areas such as South America or Africa, the annual age-adjusted incidence reaches up to 8/100,000 [1,2,3]. Incidence rises with advancing age, reaching a peak in the sixth decade of life [4]. The disease burden is highest in resource-limited settings where established risk factors are more prevalent. These are represented by phimosis, poor genital hygiene, chronic inflammation, lichen sclerosus (LS), or human papillomavirus (HPV) [5,6].

In Romania and eastern Europe more broadly, incidence of penile cancer (PC) is about 0.86–1.3/100,000, with an estimated mortality rate of 0.4/100,000, in the context of a persistent lack of region-specific evidence [2]. Regional differences in healthcare infrastructure, including variable access to specialized uro-oncological centers, absence of organized screening programs and differences in circumcision practices and HPV vaccination coverage, may influence both the stage at presentation and prognostic outcomes. Furthermore, demographic factors such as rural–urban disparities in healthcare access and socio-economic determinants may affect the timing of diagnosis and the availability of optimal surgical and adjuvant treatment. These considerations underscore the need for population-specific prognostic data, as findings from western European or South American cohorts may not be directly generalizable. Despite its rarity, the disease exerts a major impact on both survival and quality of life (QoL), causing significant psychological distress through possible sexual dysfunction and by affecting self-esteem related to genital organ involvement [7,8,9].

PSCC represents more than 95% of malignant penile tumors [10]. Patient outcomes are primarily determined by the pathological subtype, tumor grade, perineural invasion (PNI), lymphovascular invasion (LVI), extent of regional lymph node metastasis and the presence of extracapsular extension (ECE) [11,12,13]. Higher tumor grade and LVI predict metastatic dissemination, while lymphovascular space involvement is more frequently observed in advanced-stage disease [14,15].

The EAU guidelines and recent ESMO-EURACAN clinical practice guidelines indicate that lymph node involvement is the strongest prognostic factor in PC with 5-year survival rates decreasing from 90% in localized disease to about 50% in the presence of regional nodal involvement [9,16,17,18]. To predict the likelihood of LN dissemination, a series of risk scores and nomograms has been defined. However, these are not fully validated, and thus they have been excluded from current practice [19,20].

Urethral invasion remains a debated topic in penile cancer. Even if invasion of the proximal urethra may indicate aggressive disease and unfavorable prognosis, the pT2/T3 staging changes over time; its current classification (UICC/AJCC 8th edition TNM classification) is based on LVI and PNI features [21,22].

The low incidence of PSCC was directly proportional to the validity of conducting a large, prospective study, highlighting the limits of existing reports. The lack of evidence from eastern European populations is clearly pronounced [23]. To our knowledge, this represents the first comprehensive prognostic analysis from a Romanian tertiary center, contributing essential data from a region where intermediate incidence rates intersect with unique demographic and healthcare characteristics.

The aim of this paper is to address knowledge gaps—by analyzing prognostic factors in a cohort of 60 surgically treated patients with PSCC from a Romanian tertiary uro-oncology center. We hypothesized that in addition to established TNM staging components, specific pathological features—particularly urethral invasion, which is currently excluded from the AJCC 8th edition staging criteria despite its biological plausibility as a marker of aggressive disease—may independently predict overall survival (OS). The variables selected for analysis were based on established prognostic factors in the penile cancer literature (T-stage, N-stage, tumor grade, LVI, PNI, surgical margins) and clinical observations suggesting that urethral invasion may represent an underrecognized risk factor. We aimed to validate the prognostic role of established staging elements, explore the independent contribution of urethral invasion and assess whether a simplified prognostic model could be derived for clinical risk stratification.

## 2. Materials and Methods

### 2.1. Study Design and Patient Selection

This retrospective, longitudinal and observational cohort study was conducted from October 2020 to December 2024 at a single tertiary referral center for uro-oncological diseases (Uronephrology and Renal Transplant Clinic, Fundeni Clinical Institute, Bucharest, Romania). A total of 85 patients were diagnosed and underwent surgical treatment for penile tumors during this period. The study analyzed a group of 60 patients, following the application of inclusion and exclusion criteria, and received approval from the institutional ethics committee (Ethics Council of the Fundeni Clinical Institute; Decision No. 53078/2025). All procedures were performed in accordance with the 1964 Helsinki Declaration and additional amendments. The cross-sectional analysis date was December 2025. While this design ensures that all patients had a minimum of 12 months of potential follow-up, patients treated in the later period of the study (2024) had limited long-term observation compared to those treated in 2020–2022. To mitigate the impact of heterogeneous follow-up duration, restricted mean survival time (RMST) was employed alongside Kaplan–Meier estimates, providing time-limited survival summaries that are less sensitive to differential censoring.

The inclusion criteria were as follows: age ≥ 18 years at diagnosis, treatment with curative-intent surgery of primary tumor with or without lymph node dissection, and histologically confirmed PSCC and availability of complete pathological staging data. The exclusion criteria consisted of incomplete clinical, surgical, or pathological data; non-squamous histology; distant metastases (M1) at initial presentation, or palliative surgical intent.

### 2.2. Data Collection

Demographic, clinical and pathological data were extracted from electronic medical records, surgical reports, pathology databases and outpatient follow-up records. The variables collected included demographic and clinical characteristics, such as age (at diagnosis), patient comorbidities (cardiovascular disease, hypertension, body mass index [BMI] and obesity status [BMI ≥ 30 kg/m^2^]) and theoretical risk factors (phimosis, LS, smoking history, and socio-economic status–urban/rural). Socio-economic status was categorized as a binary variable (urban vs. rural residence) based on the patient’s registered domicile at the time of diagnosis, which was used as a proxy for healthcare access disparities. Smoking history was recorded as a binary variable (ever-smoker vs. never-smoker) based on self-reported data from medical records; detailed quantification of pack-years was not consistently available. We acknowledge that these binary categorizations reduce the granularity of these variables and that self-reported data from retrospective medical records are subject to reporting bias, potentially attenuating associations with outcomes.

### 2.3. Staging and Pathological Assessment

All tumors were staged according to the American Joint Committee on Cancer (AJCC) 8th edition TNM classification system [24]. Pathological specimens were evaluated by experienced uropathologists at our institution. Tumor grade was assessed using the WHO grading system. A special emphasis was placed on LVI, PNI, surgical margins and possible urethral invasion (confirmed by microscopic examination of hematoxylin and eosin-stained sections). All penectomy specimens underwent serial sectioning at 3–5 mm intervals with specific evaluation of urethral sections. Urethral invasion specifically required the documentation of one or more of the following criteria: (1) direct extension of tumor into the urethral epithelium; (2) invasion through the corpus spongiosum reaching the urethral submucosa; or (3) involvement of periurethral tissues with breach of the urethral wall integrity. The serial sectioning approach at 3–5 mm intervals may not detect all instances of microscopic urethral invasion, potentially leading to misclassification (false negatives). Additionally, while pathological specimens were evaluated by experienced uropathologists at our institution, formal interobserver agreement assessment was not performed for the identification of urethral invasion. These factors may introduce measurement error and should be considered when interpreting the prognostic significance of this variable.

An HPV status assessment was not systematically performed during the study period, as HPV testing (p16 immunohistochemistry or HPV DNA detection) was not part of routine clinical practice for PC specimens at our institution between 2020 and 2024. Consequently, HPV status data were not available for analysis.

### 2.4. Statistical Analysis

All statistical analyses were performed using R software version 4.5.2 (R Foundation for Statistical Computing, Vienna, Austria). Continuous variables were expressed as the mean ± standard deviation (SD) for normally distributed data or median for non-normally distributed data, and categorical variables were presented as absolute frequencies and percentages.

Baseline characteristics of the study population were summarized using appropriate descriptive statistics. Continuous variables were tested for normality using the Shapiro–Wilk test. Comparisons between groups were performed using Student’s *t*-test for continuous variables and chi-square or Fisher’s exact test for categorical variables, as appropriate.

OS was estimated using the Kaplan–Meier method with survival curves compared between groups using the log-rank test. For each stratum, we calculated the RMST, which represents the area under the survival curve up to a specified time point and provides a clinically interpretable measure of average survival time. Median survival with 95% confidence intervals (CIs) was also reported when the median was reached during follow-up.

Univariate Cox proportional hazards regression was performed to identify potential prognostic factors associated with OS. Hazard ratios (HRs) with 95% confidence intervals were calculated for each variable. Continuous variables (age and tumor size) were analyzed as continuous predictors. Categorical variables with more than two levels were analyzed using indicator variables with a reference category.

Variables demonstrating associations with OS at *p* < 0.10 in univariate analysis were considered for multivariate Cox regression. The *p* < 0.10 threshold, rather than conventional *p* < 0.05, was chosen to prevent the premature exclusion of potentially important prognostic factors in our limited sample (n = 60 and 28 events). With restricted statistical power, a more liberal screening threshold helps avoid errors, while subsequent rigorous multivariable selection ensures only independent predictors are retained.

Before constructing the multivariate model, we assessed multicollinearity among candidate predictors using variance inflation factors (VIFs). Variables with adjusted GVIF^(1/2) values > 1.5 were considered to demonstrate problematic multicollinearity. The final multivariate model was developed using backward stepwise selection, sequentially removing variables with the highest *p*-values while monitoring VIF values. Variables were retained in the final model if they had *p*-values < 0.10 and acceptable VIF values. The final model included only variables meeting these criteria. We acknowledge that the sample size of 60 patients with 28 events imposes constraints on multivariable modeling. The final model included three predictors with a total of five degrees of freedom, yielding an events-per-variable ratio of approximately 5.6. While this falls below the traditional recommendation of 10 events per predictor, the backward elimination approach—starting from a saturated model and sequentially removing non-significant variables while monitoring collinearity—was designed to minimize overfitting risk. The resulting three-variable model represents the most stable configuration supported by the data. Nevertheless, the limited sample size means that effect size estimates (hazard ratios) may be imprecise, as reflected in the wide confidence intervals, and the model’s generalizability requires confirmation in independent cohorts.

A two-sided *p*-value < 0.05 was considered statistically significant throughout the study. All hypothesis tests were two-sided. No adjustment for multiple comparisons was made for exploratory analyses given the hypothesis-generating nature of this retrospective study.

### 2.5. Study Endpoint

The primary endpoint was OS, which was defined as the time from the date of primary surgical treatment to death or to a specified time point (December 2025). Patients alive at the last contact were censored at that date. The secondary purpose of this study was to identify demographic, clinical, and paraclinical factors that may influence survival in these patients.

## 3. Results

### 3.1. Patient Characteristics

The final study cohort comprised data from 60 patients who underwent curative-intent surgical treatment for PSCC, in a single tertiary uro-oncology center, from October 2020 to December 2024. The median follow-up for the entire cohort was 20 months (range: 2–63 months). For patients alive at the cross-sectional analysis date (December 2025), the median follow-up was 44 months (range: 15–63 months). Baseline demographic and clinical characteristics are summarized in Table 1. The mean age at diagnosis was 62 ± 12 years. Most patients came from rural areas (55%, n = 33). Obesity was present in 25% of patients (n = 15), while hypertension affected two thirds of the cohort (67%, n = 40). Cardiovascular comorbidities were documented in 18% of patients (n = 11). Smoking history was reported in 10% (n = 6), LS was reported in 10% (n = 6), and phimosis was reported in 20% (n = 12) of patients.

### 3.2. Primary Tumor Characteristics

Regarding the surgical approach used for the primary tumor among the 60 patients, 5 (8.33%) underwent circumcision and wide tumor excision, and 14 (23.33%) were treated by glansectomy with reconstruction using a split-thickness skin graft from the abdominal flank; these patients were included in our analysis in a limited procedure category (n = 19, 32%). Of the remaining 41 patients, 36 (60%) underwent partial penectomy with or without reconstruction using a split-thickness skin graft and 5 (8%) underwent total penectomy. The choice of surgical approach was determined by tumor location and the size and extent of invasion. Organ-preserving procedures were preferred when oncologically feasible with a minimum clinical margin of 5 mm. Partial penectomy was indicated for tumors requiring more extensive resection to achieve adequate margins, while total penectomy was reserved for cases where preservation was not oncologically safe. All surgical decisions were made by a multidisciplinary tumor board that included urologic surgeons, oncologists, and pathologists (Table 2).

The mean tumor size was 37 ± 18 mm (range: 10–85 mm). Tumor ulceration or necrosis was present in 25% of cases (n = 15), and urethral invasion was documented in 15% of patients (n = 9) on pathological examination.

### 3.3. Oncological Treatment

Adjuvant chemotherapy (CHT) using paclitaxel, cisplatin, and ifosfamide (TIP) was administered to 25% of patients (n = 15); among them, seven (12%) patients received radiotherapy (RT) (Table 2). RT was administered to the primary tumor for one (1.66%) patient, and only two patients benefited from neoadjuvant CHT with TIP. The decision to initiate adjuvant therapy was made by a multidisciplinary tumor board review. Adjuvant chemotherapy (TIP regimen) was recommended for patients with pN2–N3 disease, ECE or multiple positive nodes. Adjuvant radiotherapy was considered for patients with positive or close surgical margins, extensive ECE or fixed inguinal nodes. These criteria were aligned with EAU-ASCO guideline recommendations, though individualized based on patient performance status and comorbidities.

Bilateral inguinofemoral lymph node dissection was performed in 68% of patients (n = 41), and unilateral surgery was conducted in 32% (n = 19). Pelvic lymph node dissection was performed in 22% of patients (n = 13) with confirmed inguinal node metastases, following institutional protocols, which aligns with EAU-ASCO guidelines [9].

### 3.4. Pathological Characteristics (Table 3)

Pathological T-stage distribution was conducted as follows: pT1 in 30% (n = 18), pT2 in 35% (n = 21), pT3 in 31.67% (n = 19) and pT4 in 3.33% of patients (n = 2). Tumor grade distribution was as follows: G1 (well-differentiated) in 18% (n = 11), G2 (moderately differentiated) in 48% (n = 29), and G3 (poorly differentiated) in 33% of patients (n = 20).

Pathological lymph node staging revealed the following: pN0 in 45% (n = 27), pN1 in 3% (n = 2), pN2 in 35% (n = 21) and pN3 in 17% of patients (n = 10). Overall, 55% of patients (n = 33) had lymph node metastases (pN1–3); among these node-positive patients, the mean number of positive lymph nodes was 4.2 ± 3.8 (range: 1–18).

LVI was identified in 38% of cases (n = 23), PNI was identified in 17% (n = 10) and positive surgical margins (R1) were identified in 8% of cases (n = 5). No patients had macroscopically positive margins (R2). All patients with positive margins underwent surgical revision or were managed with adjuvant RT.

**Table 3:** Pathological characteristics and TNM staging distribution.

Variable	Value
**T-stage**, n (%)	
T1	18 (30%)
T2	21 (35%)
T3	19 (31.67%)
T4	2 (3.33%)
**Tumor grade**, n (%)	
G1	11 (18.33%)
G2	29 (48.34%)
G3	20 (33.33%)
**N-stage**, n (%)	
N0	27 (45%)
N1	2 (3%)
N2	21 (35%)
N3	10 (17%)
**LVI**, n (%)	
Yes	23 (38%)
No	37 (62%)
**PNI**, n (%)	
Yes	10 (17%)
No	50 (83%)
**Surgical margins (R1)**, n (%)	
Positive	5 (8%)
Negative	55 (92%)

Data are presented as n (%). TNM staging is classified according to the AJCC 8th edition. LVI—lymphovascular invasion; PNI—perineural invasion; R1—microscopically positive margins.

### 3.5. Prognostic Factors

Univariate Cox proportional hazards regression was performed for all demographic and behavioral characteristics. Age at diagnosis showed no significant association with OS (HR = 0.99; 95% CI: 0.96–1.02; *p* = 0.704). Similarly, obesity (HR = 0.58; 95% CI: 0.26–1.28; *p* = 0.178), cardiovascular disease (HR = 0.88; 95% CI: 0.36–2.17; *p* = 0.782), hypertension (HR = 1.19; 95% CI: 0.55–2.57; *p* = 0.664), LS (HR = 0.73; 95% CI: 0.22–2.44; *p* = 0.615), phimosis (HR = 1.32; 95% CI: 0.50–3.51; *p* = 0.574), smoking status (HR = 1.18; 95% CI: 0.36–3.93; *p* = 0.784), and socio-economic status (HR = 1.03; 95% CI: 0.49–2.17; *p* = 0.944) demonstrated no significant prognostic associations (Table 4).

### 3.6. Patient Survival

**Figure 1:**
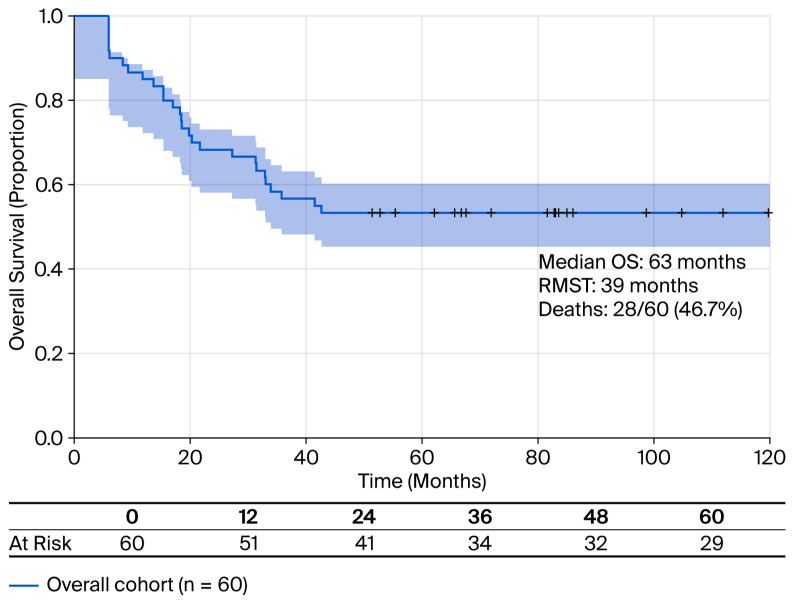
Kaplan–Meier survival curve for the entire cohort (N = 60). The median OS was 63 months with an RMST of 39 months. The shaded area represents a 95% confidence interval. During follow-up, 28 patients (46.7%) died. Tick marks indicate censored observations.

During the follow-up period, 28 out of 60 patients (46.7%) died. The median OS for the entire cohort was 63 months with a RMST of 39 months (Figure 1). The median CSS was 83.3%, but only 10 out of 28 (35.7%) were registered as cancer-specific deaths; the reason for death in the other cases was represented as cardiovascular disease (n = 7), respiratory failure (n = 4), renal failure (n = 3), sepsis (non-cancer-related deaths), and other comorbidities (n = 2).

Tumor-related factors: Tumor ulceration and necrosis showed a trend toward worse survival but did not reach statistical significance (HR = 0.52 for absence vs. presence; 95% CI: 0.23–1.16 and *p* = 0.112). Tumor size demonstrated a strong continuous association with mortality risk. Each 1 mm increase in tumor diameter was associated with a 3% increase in the risk of death (HR = 1.03; 95% CI: 1.01–1.05; *p* < 0.001).

**Figure 2:**
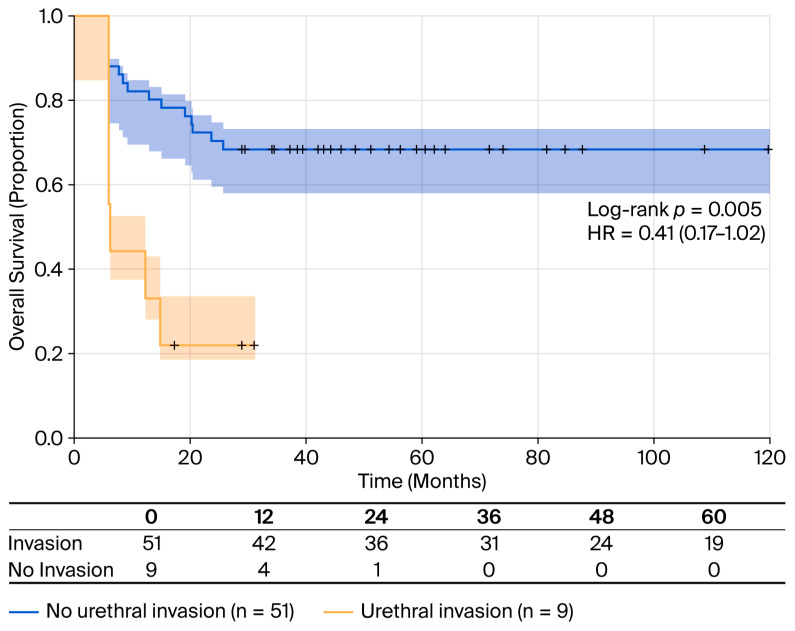
OS stratified by urethral invasion status: Kaplan–Meier survival curves comparing patients with urethral invasion (n = 9, yellow line) vs. without urethral invasion (n = 51, blue line). Patients without urethral invasion had significantly better survival outcomes (median OS 63 months; RMST 41.3 months) compared to those with urethral invasion (median OS 11 months; RMST 23.9 months). Log-rank test: *p* = 0.005. Shaded areas represent 95% confidence intervals. Tick marks indicate censored observations. HR = 0.41 (95% CI: 0.17–1.02, *p* = 0.056) for the absence vs. presence of urethral invasion.

Urethral invasion was identified on histological specimens in nine cases (two were stage pT1, two were stage pT2, four were stage pT3, and one was stage pT4). Urethral invasion is associated with substantially worse survival with absence of invasion conferring a 59% reduction in mortality risk; this effect approached but did not reach statistical significance (HR = 0.41; 95% CI: 0.17–1.02; *p* = 0.056). Patients with urethral invasion had an almost six times smaller median OS than those without invasion (11 months vs. 63 months, log-rank *p* = 0.005)—for instance, an RMST of 41.3 versus 23 months (Figure 2).

Pathological T-stage (Table 5) demonstrated a powerful dose–response relationship with OS. Compared to pT1 disease (reference category), pT2 tumors showed a non-significant trend toward worse survival (HR = 2.68; 95% CI: 0.72–9.96; and *p* = 0.142), while pT3–4 tumors presented a major 8.9-fold increased risk of death (HR = 8.90; 95% CI: 2.57–30.8; *p* < 0.001).

**Figure 3:**
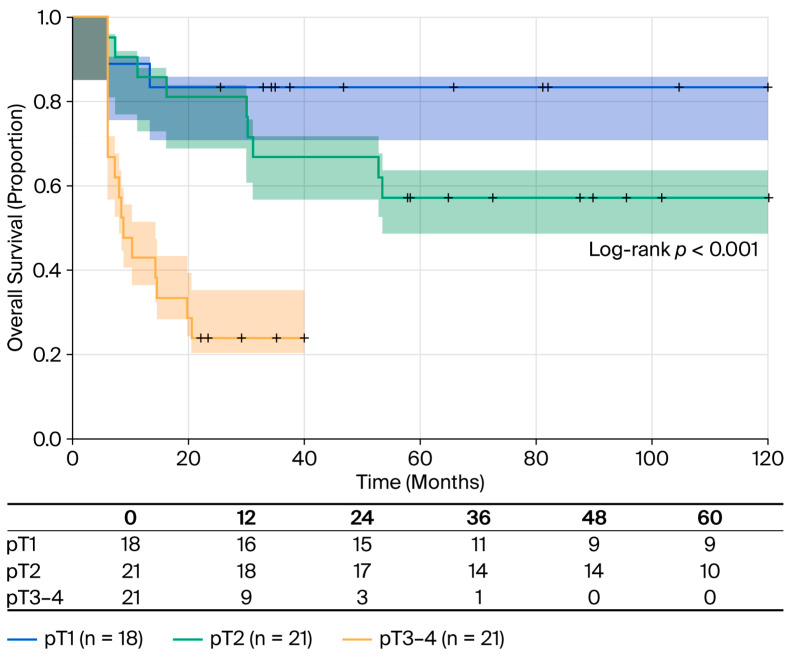
OS stratified by pathological T-stage. Kaplan–Meier survival curves stratified by pathological T-stage according to the AJCC 8th edition. pT1 (n = 18, blue line): RMST 54.4 months and 16.7% mortality (3/18); pT2 (n = 21, green line): median OS 63 months (95% CI: 17-NR), RMST 43.2 months, and 42.85% mortality (9/21); pT3–4 (n = 21, yellow line): median OS 10 months (95% CI: 6-NR), RMST 20.2 months and 76.19% mortality (16/21). Log-rank test *p* < 0.001. Shaded areas represent 95% confidence intervals. Tick marks indicate censored observations.

The corresponding RMST values for T-stage were as follows: 54.4 months for pT1, 43.2 months for pT2, and 20.2 months for pT3–4 (log-rank test: *p* < 0.001). The median OS values demonstrated the following gradient: 63 months (95% CI: 17-not reached) for pT2 and only 10 months (95% CI: 6-not reached) for pT3–4 disease. Mortality rates by T-stage were as follows: 16.7% (3/18) for pT1, 42.9% (9/21) for pT2, and 76.2% (16/21) for pT3–4 (Figure 3).

**Figure 4:**
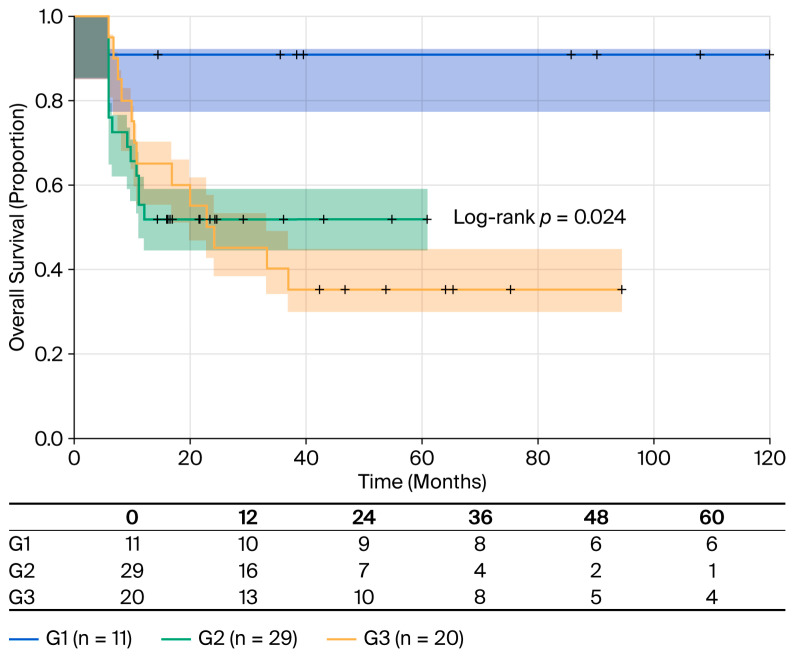
OS stratified by tumor grade: Kaplan–Meier survival curves stratified by tumor grade according to the WHO classification. G1 (n = 11, blue line): RMST of 53 months and 9.09% mortality (1/11); G2 (n = 29, green line): median OS of 19 months (95% CI: 12-NR), RMST of 34.1 months and 58.62% mortality (17/29); G3 (n = 20, yellow line): median OS of 20 months (95% CI: 9-NR), RMST of 35.6 months and 50% mortality (10/20). Log-rank test: *p* = 0.024. Shaded areas represent 95% confidence intervals. Tick marks indicate censored observations.

Tumor grade was significantly associated with OS (Table 5). Compared to G1 (well-differentiated) tumors, G2 (moderately differentiated) tumors demonstrated an eight-fold increased risk of death (HR = 8.04; 95% CI: 1.07–60.6; *p* = 0.043). By contrast, G3 (poorly differentiated) tumors showed a 7.5-fold increased risk of death, which borders on statistical significance (HR = 7.52; 95% CI: 0.96–58.8; *p* = 0.054). The wide confidence intervals reflect the small number of events in the G1 category (only one death among 11 G1 patients, resulting in 9% mortality); this limits statistical power but nevertheless demonstrates a clear trend. Median OS was not reached for G1 and (95% CI: 12-not reached) G2 at 19 months, respectively, or 20 months (95% CI: 9-not reached) for G3. RMST values were 53 months for G1, 34.1 months for G2, and 35.6 months for G3 (Figure 4).

**Figure 5:**
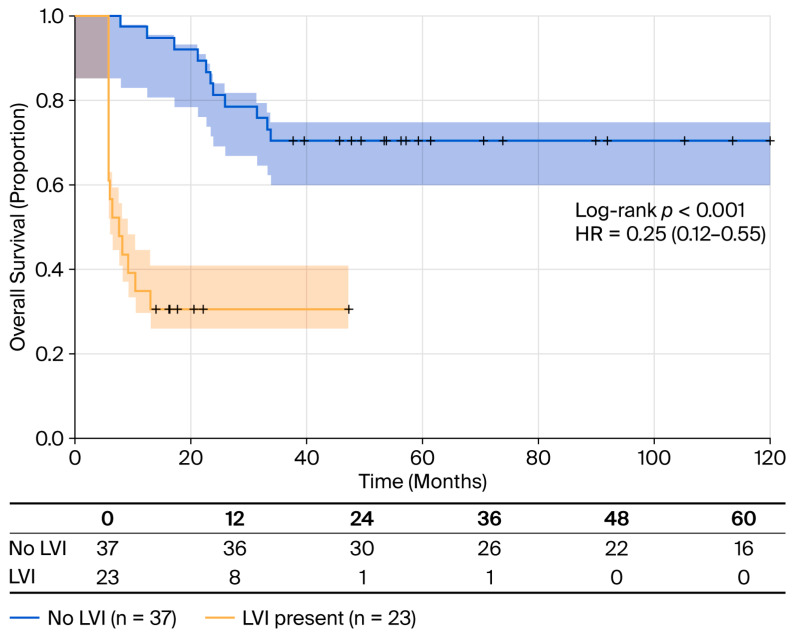
OS stratified by LVI status. Kaplan–Meier survival curves comparing patients with lymphovascular (LVI; n = 23, yellow line) and without lymphovascular invasion (n = 37, blue line). Patients without LVI had significantly better survival (RMST 45.8 months) compared to those with LVI (RMST 19.4 months). Mortality rates: 32.43% (12/37) without LVI versus 69.56% (16/23) with LVI. Log-rank test: *p* < 0.001. Shaded areas represent 95% confidence intervals. Tick marks indicate censored observations. HR = 0.25 (95% CI: 0.12–0.55, *p* < 0.001) for absence versus presence of LVI.

The absence of LVI has been shown to be a protective factor, as it indicates a 75% reduction in mortality risk (HR = 0.25; 95% CI: 0.12–0.55; *p* < 0.001) (Table 5). Patients without LVI did not reach median OS (95% CI: 63 months—not reached) compared to an OS of 11 months (95% CI: seven—not reached) for those with LVI. Mortality rates were 32.4% (12/37) without LVI versus 69.6% (16/23) with LVI (log-rank *p* < 0.001) (Figure 5).

**Figure 6:**
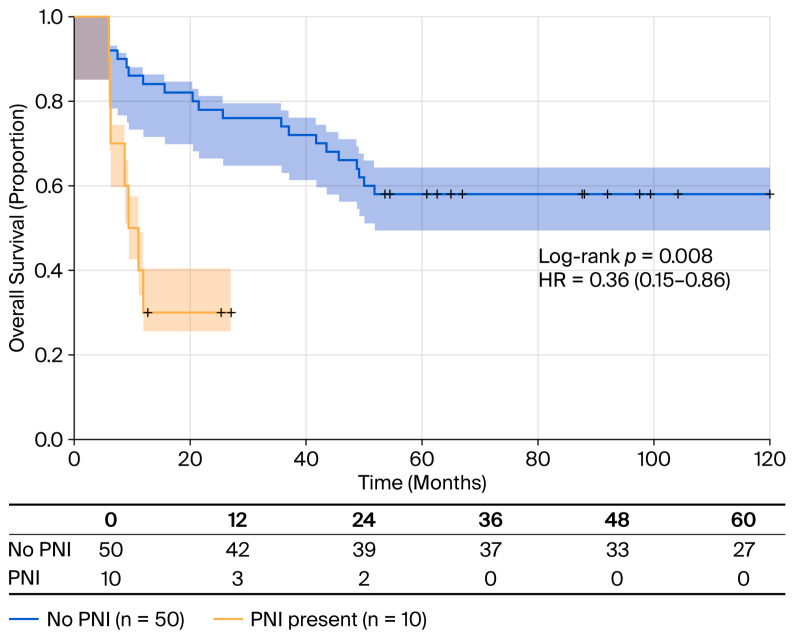
OS stratified by PNI status. Kaplan–Meier survival curves comparing patients with perineural invasion (PNI; n = 10, yellow line) to those without PNI (n = 50, blue line). Patients without PNI had significantly better survival (median OS 63 months; 95% CI: 20-NR; and RMST 41.9 months) compared to those with PNI (median OS 10 months; 95% CI: 6-NR; and RMST 23.4 months). Mortality rates: 42.0% (21/50) without PNI versus 70.0% (7/10) with PNI. Log-rank test *p* = 0.008. Shaded areas represent 95% confidence intervals. Tick marks indicate censored observations. HR = 0.36 (95% CI: 0.15–0.86, *p* = 0.022) for absence versus presence of PNI.

The absence of PNI was a similar protective factor, indicating a 64% reduction in mortality risk (HR = 0.36; 95% CI: 0.15–0.86; *p* = 0.022) (Table 5). The median OS was 63 months (95% CI: 20-not reached) for patients without PNI compares to 10 months (95% CI: six-not reached) for those with PNI (Figure 6). Mortality rates were 42.0% (21/50) without PNI versus 70.0% (7/10) with PNI (log-rank test: *p* = 0.008).

**Figure 7:**
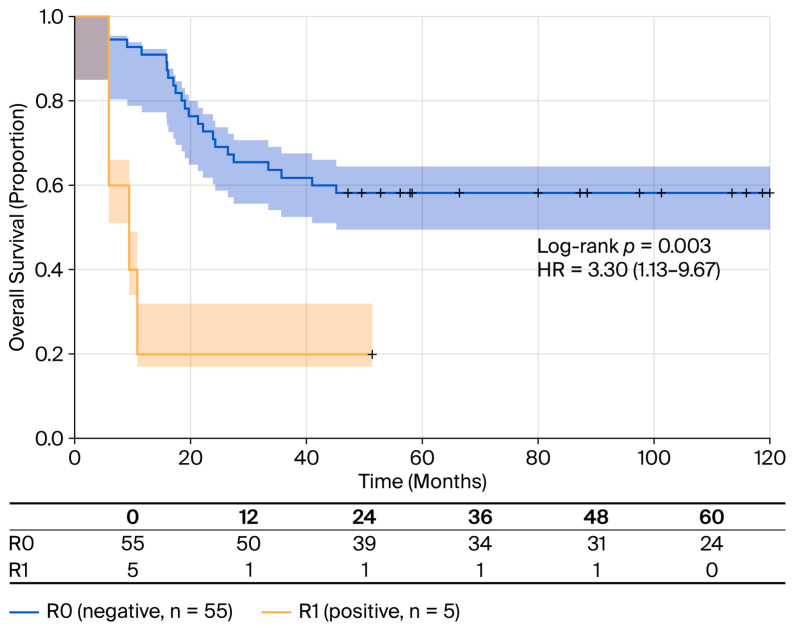
OS stratified by surgical margin status. Kaplan–Meier survival curves comparing patients with positive surgical margins (R1; n = 5, yellow line) and negative margins (R0; n = 55, blue line). Patients with R0 resection had significantly better survival outcomes (median OS 63 months; 95% CI: 19 - NR; and RMST 40.8 months) compared to those with R1 resection (median OS 10 months; 95% CI: 7 - NR; and RMST 15.4 months). Mortality rates: 43.63% (24/55) for R0 versus 80.0% (4/5) for R1. Log-rank test: *p* = 0.003. Shaded areas represent 95% confidence intervals. Tick marks indicate censored observations. HR = 3.30 (95% CI: 1.13–9.67; *p* = 0.030) for R1 compared to R0.

Positive surgical margins (R1) were associated with a 3.3-fold increased risk of mortality (HR = 3.30; 95% CI: 1.13–9.67; *p* = 0.030) compared to negative margins (R0) (Table 5). Patients with positive margins had a median OS of only 10 months (95% CI: seven-not reached) compared to 63 months (95% CI: 19-not reached) for those with negative margins (Figure 7). Mortality rates were 43.6% (24/55) for R0 versus 80.0% (4/5) for R1 (log-rank test: *p* = 0.003).

**Figure 8:**
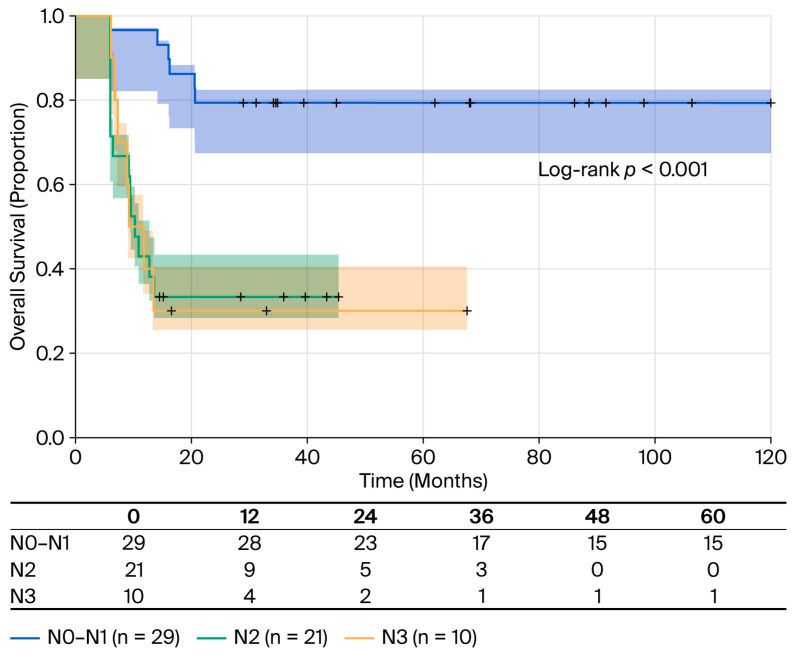
OS stratified by pathological nodal stage. Kaplan–Meier survival curves stratified by pathological nodal stage according to the AJCC 8th edition. N0–N1 (n = 29, blue line): RMST of 52.3 months and 24.13% mortality (7/29); N2 (n = 21, green line): median OS of 12 months (95% CI: 9-NR), RMST of 27.7 months and 66.67% mortality (14/21); N3 (n = 10, yellow line): median OS of 13.5 months (95% CI: 6-NR), RMST of 23.8 months and 70.0% mortality (7/10). Log-rank test: *p* < 0.001. Shaded areas represent 95% confidence intervals. Tick marks indicate censored observations. Compared to N0–N1, N2 had HR = 4.07 (95% CI: 1.64–10.1, *p* = 0.003) and N3 had HR = 4.78 (95% CI: 1.66–13.8, *p* = 0.004).

Univariate Cox regression analysis revealed significant associations between N-stage and mortality risk (Table 6). Using N0–N1 as a reference (due to there being only a small number of patients with N1), patients with N2 disease had a 4.07-fold increased risk of death (HR 4.07; 95% CI 1.64–10.1; *p* = 0.003), while N3 patients exhibited a 4.78-fold increased risk (HR 4.78; 95% CI 1.66–13.8; *p* = 0.004). Kaplan–Meier survival analysis (Figure 8) showed marked differences in outcomes across N-stages. The N0–N1 cohort (n = 29) had a mortality rate of 24.13% (7/29 deaths) with an RMST of 52.3 months and did not reach median survival (95% CI: 63.00 to not estimable value). In contrast, N2 patients (n = 21) experienced 66.67% mortality (14/21 deaths), an RMST of 27.7 months, and a median survival of 12 months (95% CI: 9.00 to not estimable value), and the N3 group (n = 10) demonstrated 70% mortality, an RMST of 23.8 months, and a median survival of 13.5 months.

In the statistical analysis, patients were grouped according to their indicated stage or curative lymph node dissection (LND) according to the guidelines of the European Association of Urology. The following categories were used: C1 patients without an indication for LND; C2 patients with an indication for LND and early LND considered; C3 patients with an indication for LND with delayed LND considered; and C4 patients with an indication for LND, which was not performed (due to a lack of compliance). Cox regression analysis of the timing for second-stage surgery revealed significant survival implications (Table 6). Compared to patients without indication for second-stage surgery (C1, reference group), those who underwent early second-stage surgery (C2) demonstrated a substantially elevated hazard ratio (HR 9.93; 95% CI 1.31–75.6; *p =* 0.027). Patients with delayed surgery (C3) showed a non-significant trend toward increased risk (HR 5.05; 95% CI 0.53–48.6; *p =* 0.161), and those who did not undergo indicated surgery (C4) demonstrated borderline significance (HR 7.27; 95% CI 0.92–57.5; *p =* 0.060).

**Figure 9:**
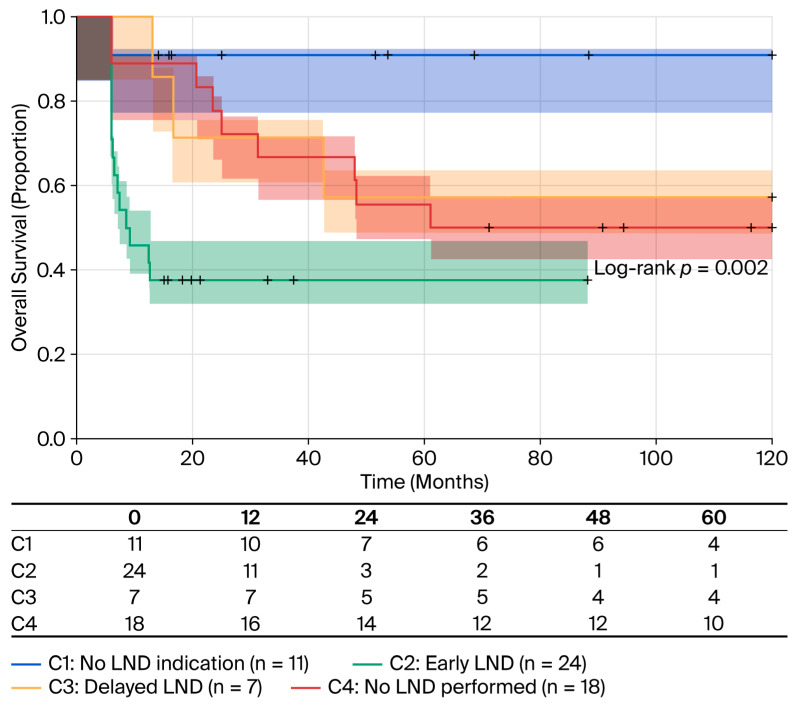
OS stratified by timing and indication of second-stage surgery. Kaplan–Meier survival curves stratified by lymph node dissection (LND) and indication and timing of surgery according to EAU guidelines. C1, no indication for LND (n = 11, blue line): RMST of 53.9 months and 9.09% mortality (1/11); C2, LND performed early (n = 24, green line): median OS of 12.5 months (95% CI: 11-NR), RMST of 29.3 months and 62.5% mortality (15/24); C3, delayed LND (n = 7, yellow line): RMST of 39.9 months and 42.85% mortality(3/7); C4, LND indicated but not performed (n = 18, red line): median OS of 63 months (95% CI: 12-NR), RMST of 38.3 months and 50.0% mortality (9/18). Log-rank test: *p* = 0.002. Shaded areas represent 95% confidence intervals. Tick marks indicate censored observations. Compared to C1, C2 HR = 9.93 (95% CI: 1.31–75.6; *p* = 0.027), C3 HR = 5.05 (95% CI: 0.53–48.6; *p* = 0.161), C4 HR = 7.27 (95% CI: 0.92–57.5; *p* = 0.060).

Kaplan–Meier analysis highlighted survival disparities across groups (Figure 9). The C1 cohort (n = 11) exhibited the most favorable prognosis with 9.09% mortality and an RMST of 53.9 months. The C2 group (n = 24) experienced 62.5% mortality, an RMST of 29.3 month, and a median survival of 12.5 months (95% CI: 11.00 to not estimable value). Patients with delayed surgery (C3, n = 7) had 42.85% mortality (3/7 deaths), an RMST of 39.9 months, and did not reach median survival (95% CI: 17.00 to not estimable value). The C4 cohort (n = 18) demonstrated 50% mortality, an RMST of 38.3 months, and a median survival of 63 months.

### 3.7. Multivariate Analysis: Independent Prognostic Factors

Variables demonstrating associations with OS at *p* < 0.10 in univariate analysis were entered into multivariate Cox regression modeling. These included tumor size, urethral invasion, pathological T-stage, tumor grade, PNI, LVI, surgical margins, pathological N-stage and second-stage surgery status (Table 7).

Variance inflation factor (VIF) analysis revealed moderate multicollinearity with adjusted GVIF ranging from 1.2 to 1.5 across predictors. Second-stage surgery demonstrated the highest GVIF (4.5, adjusted GVIF 1.3), while LVI had the lowest GVIF (1.6, adjusted GVIF 1.3). In the initial model, only T3–T4 stages reached statistical significance (HR 5.32, 95% CI 1.06–26.7, *p* = 0.042 vs. T1).

**Table 7:** Initial multivariate Cox regression model including all candidate predictors.

Variable	Total (N)	Deaths (N)	HR (95% CI)	*p*-Value	GVIF	Adjusted GVIF
**Tumor size** (mm)	60	28	1.01 (0.98–1.04)	0.562	2.1	1.5
**Urethral invasion**					2.3	1.5
Yes	9	6	-			
No	51	22	0.35 (0.08–1.51)	0.160		
**T-stage**					3.2	1.3
T1	18	3	-			
T2	21	9	1.66 (0.33–8.33)	0.537		
T3–T4	21	16	5.32 (1.06–26.7)	0.042		
**Tumor grade**					1.9	1.2
G1	11	1				
G2	29	17	4.93 (0.56–43.7)	0.151		
G3	20	10	4.28 (0.44–42.1)	0.213		
**PNI**					1.8	1.3
Yes	10	7	-			
No	50	21	1.22 (0.37–4.02)	0.748		
**LVI**					1.6	1.3
Yes	23	16	-			
No	37	12	0.46 (0.17–1.26)	0.132		
**Surgical margins**					2.2	1.5
Negative (R0)	55	24	-			
Positive (R1)	5	4	0.67 (0.14–3.26)	0.618		
**N-stage**					3.0	1.3
N0–1	29	7	-			
N2	21	14	0.77 (0.18–3.22)	0.715		
N3	10	7	2.44 (0.63–9.50)	0.198		
**LND timing**					4.5	1.3
C1	11	1	-			
C2	24	15	1.43 (0.09–23.30)	0.802		
C3	7	3	0.92 (0.05–18.70)	0.959		
C4	18	9	1.00 (0.05–21.60)	0.999		

HR, hazard ratio; CI, confidence interval; GVIF, generalized variance inflation factor; LVI, lymphovascular invasion; PNI, perineural invasion; LND, lymph node dissection. The model includes all variables with *p* < 0.10 in univariate analysis. Adj. GVIF^(1/2) values > 1.5 indicate problematic multicollinearity.

**Figure 10:**
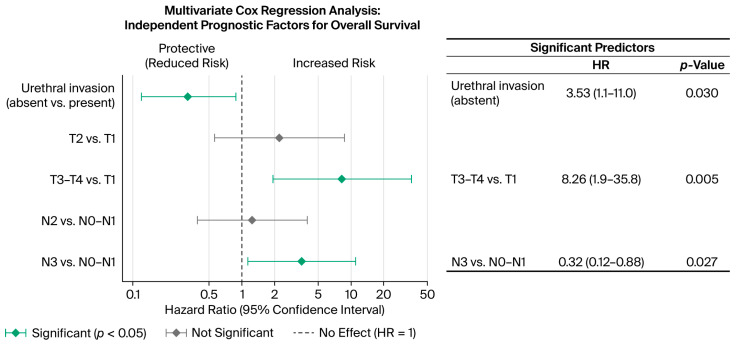
Multivariate Cox regression analysis of prognostic factors for OS. Forest plot displays hazard ratios (HRs) with 95% confidence intervals (CIs) from multivariate Cox proportional hazards regression analysis. Diamond markers represent HR point estimates; horizontal lines show a 95% CI. Green indicates statistically significant independent predictors (*p* < 0.05); gray indicates non-significant variables. A dashed vertical line at HR = 1.0 represents no effect. The right panel summarizes three significant predictors: absence of urethral invasion (HR = 0.32; *p* = 0.027), T3–T4 stages (HR = 8.26; *p* = 0.005), and N3 disease (HR = 3.53; *p* = 0.030). The *X*-axis is presented on a logarithmic scale.

Backward selection based on *p*-values and VIF metrics yielded a parsimonious model with three independent predictors: urethral invasion, T-stage, and N-stage (Figure 10). The refined model exhibited improved collinearity diagnostics (adjusted GVIF: 1.1) while maintaining adequate statistical power (Table 8).

Urethral invasion has been identified as a significant prognostic factor. Patients without urethral invasion demonstrated a 68% reduction in mortality compared to those with invasion (HR 0.32; 95% CI 0.12–0.88; *p* = 0.027).

T-stage exhibited a dose–response relationship with mortality risk. Relative to T1 disease (reference), T2 patients had a non-significant trend toward increased risk (HR 2.20; 95% CI 0.56–8.69; *p* = 0.262), whereas T3–T4 stages of disease conferred an 8.26-fold increase in mortality risk (HR 8.26; 95% CI 1.91–35.8; *p* = 0.005).

N-stage demonstrated prognostic stratification in advanced disease. Compared to N0–N1 (reference), N2 patients exhibited no significant difference in mortality (HR 1.24; 95% CI 0.40–3.86; *p* = 0.716), while N3 disease was associated with a 3.53-fold increased risk (HR 3.53; 95% CI 1.13–11.0; *p* = 0.030).

The final multivariate model demonstrated good discriminative ability, with a Harrell’s C-index of 0.78 (95% CI: 0.69–0.87), indicating that the model correctly ranked patient survival times in 78% of patient pairs.

## 4. Discussion

This retrospective cohort study of 60 patients surgically treated for PSCC at a Romanian tertiary uro-oncology center is the first national report of its kind. Studying the diagnosis and surgery used to treat penile tumors contributes to the limited literature on PC for an underrepresented region (central and eastern Europe). The data validate core TNM staging components while identifying urethral invasion as an important high-risk prognostic factor, warranting recognition in clinical practice and potentially in future staging revisions.

### 4.1. Demographic and Clinical Factors

Our finding is that clinical and demographic characteristics are not associated with the published literature emphasizing tumor biology over host factors in determining PSCC prognosis [24,25]. Thus, age showed no association with survival (HR = 0.99 per year, *p* = 0.704), which is consistent with SEER data, demonstrating that while PC incidence increases with age, survival outcomes are primarily stage dependent [25].

The lack of correlation between established risk factors (phimosis HR = 1.32 and *p* = 0.574; smoking HR = 1.18 and *p* = 0.784) and survival may suggest that these factors are determinants in carcinogenesis but not necessarily negative prognostic factors once cancer develops. Contrary to the literature [24], our data indicate that these characteristics do not independently influence survival outcomes in surgically treated patients. Similarly, comorbidities including cardiovascular disease (HR = 0.88; *p* = 0.782) and hypertension (HR = 1.19; *p* = 0.664) showed no prognostic impact. These results could lead to the exclusion of patients with prohibitive operative risk from surgical treatment; second, they suggest the limited impact of comorbidities on PSCC survival in accordance with histopathological characteristics.

### 4.2. Key Findings in Context

This paper demonstrates a clear dose–response relationship between T-stage and mortality risk. The distribution of T-stages in this paper was 30% pT1, 35% pT2, 31.67% pT3, and 3.33% pT4, indicating that most patients presented with invasive disease at diagnosis. By referring to T1 disease, T2 patients showed a non-significant trend toward increased risk (HR 2.20; 95% CI 0.56–8.69; *p* = 0.262), whereas T3–T4 disease conferred an 8.26-fold increase in mortality risk (HR 8.26; 95% CI 1.91–35.8; *p* = 0.005) with a median OS of only 10 months. Similarly, a recent study demonstrated significant differences in OS between T2 and T3 stages (*p* = 0.03) and between T3 and T4 stages (*p* = 0.01) [26], confirming the prognostic importance of T-staging across different European populations.

Nodal disease was validated as the strongest prognostic factor. Our finding that pN3 disease confers a 3.5-fold increased risk of mortality (HR = 3.53; *p* = 0.030) validates the extensive literature establishing nodal metastases as the most significant prognostic determinant of PSCC. The ESMO-EURACAN guidelines report a significant decrease in 5-year survival from 90% in localized disease to 50% with lymph node involvement [9]. Our data validate this assertion with survival clearly separated by nodal burden. The survival outcomes demonstrate relatively similar values to those in the literature: 76% for pN0/N1, 33% for pN2, and 30% for pN3, in comparison with a 5-year CSS of 95%, 80%, 65%, and 35% for N0, N1, N2, and N3 disease, respectively [11,27]. An important observation is that pN2 disease did not achieve independent significance in our multivariate model (HR = 1.24; *p* = 0.716) when adjusted for T-stage, whereas pN3 retained significance. This may suggest that bulky nodal disease often coexists with locally advanced primary tumors.

The independent prognostic significance of urethral invasion (HR = 0.32 for absence; *p* = 0.027) represents an important finding that could have potential in future classifications. Patients without urethral invasion had a median OS of 63 months versus only 11 months for those with urethral invasion—a 6-fold difference that persisted after adjusting for T- and N-stages. These results are contradictory to the UICC 8th, which does not consider urethral invasion as a prognostic factor. However, at the same time, it states that invasion of the proximal urethra may represent more aggressive PSCC with a poor prognosis [21]. Urethral invasion’s impact on survival was explored by Campos et al., who analyzed 125 patients who underwent penile amputation and bilateral LND. Lymph node metastasis (RR = 57.9; 95% CI = 7.4 to 453.9), urethral infiltration (RR = 3.5; 95% CI = 1.3 to 9.2) and MMP-9 immunoreactivity (RR = 3.2; 95% CI = 1.2 to 8.3) were identified as independent risk factors for disease recurrence on multivariate analysis [28]. In contrast to our data, Zequan et al. [29] analyzed urethral invasion in PSCC in a group of 101 patients and found no independent association with survival in the multivariate model. These differences may reflect variations in the histopathological definition of urethral invasion, patient inclusion criteria, and adjustments for confounders [29].

The biological plausibility of urethral invasion as a prognostic marker is supported by several mechanisms: (1) early lymphatic dissemination through adjacent lymphovascular plexus; (2) more aggressive histological subtypes in terms of dissemination; (3) surgical complexity limiting margin-negative resection; and (4) potential for occult disease extension. From a clinical standpoint, these findings suggest several practical implications: a reconsideration of staging with potential implications for adjuvant treatment in the case of urethral involvement; more extensive surgical resections in the case of previously documented urethral involvement; and routine assessment of urethral invasion in histopathological reports.

We acknowledge that the three independent predictors identified (T-stage, N-stage and urethral invasion) are biologically interrelated. Urethral invasion may be associated with more advanced T-stages, and advanced T-stages often coexist with nodal disease. To address this concern, multicollinearity was formally assessed using the variance inflation factors (VIF). The final model demonstrated low collinearity (adjusted GVIF values of 1.1 for all three predictors), indicating that each variable contributes unique prognostic information beyond what is captured by the others. Furthermore, urethral invasion was observed across a range of T-stages in our cohort (2 pT1, 2 pT2, 4 pT3, and 1 pT4), suggesting that it is not merely a surrogate for advanced local staging. Nevertheless, the biological correlation between these factors is recognized, and future larger studies should explore potential interactions and assess whether urethral invasion adds prognostic value beyond the T-stage in multivariable models with greater statistical power.

A notable finding is that only 35.7% of deaths (10/28) were attributable to PC progression, with the majority (64.3%, 18/28) resulting from other causes (cardiovascular disease, respiratory failure, renal failure or other comorbidities). This reflects the substantial competing mortality risk in our cohort with a high prevalence of cardiovascular risk factors and smoking history. The divergence between CSS: (83.3%) and OS (53.3%) underscores the importance of distinguishing disease-specific and all-cause mortality when counseling patients and interpreting survival outcomes in PSCC.

### 4.3. Histopathological Features

We identified factors that demonstrated a strong association with survival in univariate analysis but did not retain independence in multivariate analysis. This pattern provides biological and clinical relevance, but its prognostic impact is inferior to histopathological staging elements.

The absence of LVI conferred a 75% mortality reduction in univariate analysis (HR = 0.25, *p* < 0.001), but this value lost significance in multivariate modeling. This likely reflects that LVI is both a mechanism and marker of nodal metastases. Similarly, PNI showed a strong univariate association (HR = 0.36, *p* = 0.022) but not independence, and the tumor grade demonstrated univariate associations (G2 vs. G1: HR = 8.04, *p* = 0.043) but not significance in multivariate analysis.

These characteristics have been the basis for defining risk scores in the literature. The Chaux prognostic index focuses on grade, anatomical level, and PNI [19]. By contrast, Sali et al. proposed a histopathological risk score-based pattern of infiltration, grade and anatomical level of involvement [20]. The Chaux index is a tool intended to predict nodal metastases and 5-year survival, and our findings validate the importance of these components [19]. Grade is incorporated in both risk stratification scores and forms a component of the ICCR dataset for penile carcinoma [9]. The strong relationship between grade and survival is structured on associations with both T- and N-stages—a high tumor grade correlates with advanced disease at presentation or some histological subtypes [14,15]. This paper’s identification of T-stage, N-stage, and urethral invasion as independent predictors suggests the potential for a simplified prognostic framework based on three available clinical/pathological variables. This can offer practical advantages over more complex indices requiring specialized pathological assessment or scoring calculations. However, this model should be considered preliminary and hypothesis generating. The C-index of 0.78 (95% CI: 0.69–0.87) indicates good but not excellent discrimination, and the confidence interval reflects uncertainty inherent to the sample size. Internal validation through bootstrapping was not performed in this paper, representing an additional limitation. External validation in an independent cohort—ideally from another penile cancer referral center—is essential before this model can be recommended for clinical use. We plan to pursue multicenter collaboration with other eastern European penile cancer centers to facilitate this validation.

Positive surgical margins (R1) were associated with a 3.3-fold increased risk of mortality (HR = 3.30; 95% CI: 1.13–9.67; *p* = 0.030), but these results were not independent. Although the guidelines mention that there is no clear evidence regarding the optimal width of macroscopic negative surgical margins [30,31], it is recommended to adopt extended resection techniques or partial penectomy for tumors with an advanced T-stage or a high grade, and patients should be counseled about individual risk profiles.

### 4.4. Considerations for Surgical Treatment

Guidelines support penile-preserving techniques when oncologically appropriate. A balance must be maintained between oncological outcome and technical feasibility, depending on the size and relationship to adjacent structures. The advantages are represented by the preservation or minimal impairment of erectile function and the maintenance of a good QoL both physically and psychologically. Several studies support the fact that 85–100% of men can achieve an erection and maintain their sexual function after penile-preserving surgery [32,33,34,35]. Kieffer et al. evaluated the impact of primary surgery technique and lymphadenectomy on sexuality and QoL; they reported significantly more problems in the partial penectomy group than the penile-sparing cohort, including orgasm (effect size 0.54, *p* = 0.031), appearance concerns (effect size 0.61, *p* = 0.008), life interference (effect size 0.49, *p* = 0.032) and urinary function (83% vs. 43%, *p* < 0.0001) [7].

Health-related QoL was assessed by Sosnowski et al. in a cohort of 51 patients treated for PC and found a significant negative correlation between aggressiveness of the surgical procedure and both assessment of global health status (*p* = 0.04) and physical functioning (*p* = 0.047) [36]. This hypothesis is not entirely supported by Wan et al., who evaluated the QoL parameters in a group of 15 patients with incipient penile tumors who underwent either wide local excision or partial penectomy. No statistically significant differences were observed between these two types in the data collected via the IIEF-15, SEAR, EDITS, and EORTC-QLQ-C30 questionnaires and urodynamic determinations (*p* > 0.05) except for orgasmic function (*p* = 0.033) [37].

The centralization of rare tumor pathologies, such as PC, can result in an 80% rate of preservation procedures in countries such as the UK [24]. At the national level, the Fundeni Clinical Institute is a specialized center that centralizes this pathology, generally for advanced forms. In our investigation, organ sparing was achieved in 31.67% of patients, partial penectomy was achieved in 60%, and total penectomy was achieved in 8.33%.

For nodal management, the guidelines describe two scenarios. In cases with clinically node-negative groins (cN0), early LND is recommended depending on the histopathology of the primary tumor. The impact on survival outcomes is sustained by Kroon et al., who compared early and delayed LND in a total of 40 patients staged T2–3N0. The disease-specific 3-year survival of patients with delayed LND was 35%, and in those who underwent early resection, survival rates were 84% (log rank test: *p* = 0.0017) [38]. The second scenario is represented by cN+ patients, in which radical LND is the standard of care due to a 45–80% risk of nodal metastases [39].

In this paper, the nodal metastasis rate was 58.33%, and 31 patients (63.26% among those with indication) underwent bilateral modified or radical inguinofemoral LND. The main reasons for not performing NLD among all patients with an indication of risk were lack of compliance with the proposed treatment program, and a few cases had limited access to the hospital due to the COVID-19 pandemic. The rate of non-adherence to international guidance in terms of LND is about 26.3% [40,41], and Cindolo et al. report that this leads to a statistically significant association with OS (adjusted HR: 0.48; 95% CI: 0.24–0.96; *p* = 0.038) for patients managed with LND [42].

A paradoxical finding was the similarity of survival between patients with delayed LND (C3; n = 7 and 42.85% mortality) and those with unperformed LND despite indication (C4; n = 18 and 50% mortality). This outcome may be due to several factors: First, the C3 group comprises only seven patients, resulting in statistically unstable values (HR = 5.05; 95% CI: 0.53–48.6; *p* = 0.161) with confidence intervals spanning nearly two orders of magnitude. Second, among the patients in group C4, the main reason for unperformed LND was a lack of patient compliance, potentially including patients with favorable cancer biology who remained clinically stable without intervention. Third, delayed LND in bulky nodal disease may increase surgical morbidity without survival benefit—one patient from the C3 group died from post-operative sepsis within 90 days. While these findings suggest that delayed LND may not confer a survival benefit, the small sample sizes (n = 7, respectively n = 18) and wide confidence intervals preclude definitive conclusions. Multi-institutional collaborative studies are needed to adequately address this question.

### 4.5. Limitations

Several limitations must be considered when interpreting our findings. The retrospective design precluded standardized follow-up protocols, potentially introducing selection or information bias. The therapeutic approach was made based on patient characteristics rather than randomization, potentially introducing confounding by indication. At the same time, the rarity of this pathology is recognized and limits the possibility of conducting large, prospective studies. Furthermore, the inclusion of only patients who underwent curative-intent surgery with complete pathological data introduces a selection bias that excludes the most advanced or aggressive presentations—patients who were deemed inoperable, received palliative care only, or had incomplete pathological assessment. This means our cohort represents a potentially more favorable subset of the overall PSCC population, and our survival estimates and prognostic associations may not be generalizable to all PSCC patients, particularly those with unresectable disease.

An important limitation is the absence of HPV status assessment, as HPV testing was not routinely performed during the study period. HPV-positive tumors may exhibit distinct prognostic characteristics, and the lack of HPV data prevents the stratification of our cohort by viral status. This limitation is particularly relevant for eastern European populations where HPV prevalence in PC remains poorly characterized. Future prospective studies from our center will incorporate routine p16 immunohistochemistry and HPV DNA testing to enable prognostic stratification by viral status, which is increasingly recognized as an important variable in penile cancer biology and treatment response.

The pathological assessment of urethral invasion, while based on standardized serial sectioning and defined criteria, may be subject to detection limitations (3–5 mm intervals may miss microscopic foci) and interobserver variability, as formal concordance studies were not performed.

Even though our cohort (N = 60) exceeds several single-center patient series reported to date in the literature, the sample size with 28 events limited our ability to detect modest effect sizes, particularly for rare events. The events-per-variable ratio in the final model (approximately 5.6) falls below the traditional recommendation of 10, raising the possibility of overfitting and unstable HR estimates. The wide CI observed throughout our analysis reflects this limitation. The use of backward stepwise selection, VIF monitoring and a parsimonious final model represent our efforts to mitigate these risks, but they cannot fully eliminate them. These findings should therefore be regarded as hypothesis generating and require confirmation in larger, multicenter cohorts. Additionally, although multicollinearity diagnostics (VIF) were satisfactory in our final model, the biological interrelation between T-stage, N-stage and urethral invasion is acknowledged. In larger cohorts, interaction terms and stratified analyses could more definitively establish the independence of these predictors.

We acknowledge that the examination of multiple prognostic variables without formal correction for multiplicity increases the risk of type I errors (false-positive associations). This is an inherent limitation of exploratory survival analyses in rare tumors with limited sample sizes, where overly conservative correction methods risk obscuring clinically meaningful associations. All univariate associations should therefore be interpreted as hypothesis generating with confirmatory value reserved for the variables that retained independence in multivariable analysis.

The follow-up duration was heterogeneous across the cohort, with patients treated in late 2024 having a minimum of approximately 12 months of observation, while those treated earlier had up to 62 months of follow-up. These differentials limit the maturity of long-term survival estimates, particularly for patients enrolled in the most recent period. RMST was employed to provide time-limited survival summaries that are less sensitive to late censoring, but definitive long-term survival conclusions require extended follow-up. Continued longitudinal monitoring of this cohort is planned to provide mature survival data.

An indirect limitation of the results is represented by the narrow range of oncological therapeutic options through national programs. Our prognostic model (C-index 0.78) requires validation in independent external cohorts before clinical deployment. Internal validation through resampling methods (e.g., bootstrapping) was not performed, which represents an additional limitation. Prospective multicenter validation is planned to assess the model’s generalizability across different populations and healthcare settings.

## 5. Conclusions

This retrospective cohort study of 60 patients surgically treated for penile squamous cell carcinoma identifies three independent prognostic factors: pathological T-stage, N-stage, and urethral invasion. The independent prognostic value of urethral invasion represents a key finding with clinical implications. Despite the UICC 8th edition’s statement that urethral invasion is not prognostic, our data demonstrate a six-fold survival difference (median OS of 63 vs. 11 months) that persists after adjusting for T- and N-stage. While LVI, PNI, tumor grade, and surgical margins showed strong univariate associations, their prognostic information appears to largely highlight pathological stage. Nevertheless, comprehensive pathological assessment following datasets remains essential for risk stratification and treatment planning.

Unfavorable outcomes or urethral invasion highlight the inadequacy of current treatments for advanced disease and support the enrollment of novel systemic agents, including checkpoint-targeted therapies, in clinical trials. These findings from a Romanian tertiary center validate international guideline-cited prognostic factors in an eastern European population, contribute novel data on the independent value of urethral invasion, and provide preliminary evidence for risk-adapted treatment strategies in PSCC. The three-variable prognostic model (C-index 0.78) represents a hypothesis-generating framework that requires validation in independent external cohorts before clinical deployment. Multicenter collaborative studies, ideally involving other eastern European and international penile cancer centers, are needed to confirm these findings and refine risk stratification tools for this rare malignancy.

## Figures and Tables

**Table 1:** Baseline demographic and clinical characteristics of the study cohort.

Variable	Value
**Age** (years), mean ± SD	62 ± 12
**Socio-economic status**, n (%)	
Urban	27 (45%)
Rural	33 (55%)
**Obesity**, n (%)	
Yes	15 (25%)
No	45 (75%)
**Hypertension**, n (%)	
Yes	40 (67%)
No	20 (33%)
**Cardiovascular comorbidities**, n (%)	
Yes	11 (18%)
No	49 (82%)
**Smoking history**, n (%)	
Yes	6 (10%)
No	54 (90%)
**Lichen sclerosus**, n (%)	
Yes	6 (10%)
No	54 (90%)
**Phimosis**, n (%)	
Yes	12 (20%)
No	48 (80%)

Data are presented as n (%) for categorical variables and mean ± SD or median (range) for continuous variables.

**Table 2:** Primary tumor surgical approach and oncological treatment characteristics.

Variable	Value
**Primary Tumor Surgery**, n (%)	
Circumcision + wide excision	5 (8.34%)
Glansectomy with reconstruction	14 (23.33%)
Limited procedures (total)	19 (31.67%)
Partial penectomy	36 (60%)
Total penectomy	5 (8.33%)
**Tumor size** (mm), mean ± SD (range)	37 ± 18 (10–85)
**Ulcerations/Necrosis,** n (%)	
Yes	15 (25%)
No	45 (75%)
**Urethral invasion**, n (%)	
Yes	9 (15%)
No	51 (85%)
**Adjuvant treatment**, n (%)	
Yes	15 (25%)
No	45 (75%)

Data are presented as n (%). Limited procedures include circumcision with wide tumor excision and glansectomy.

**Table 4:** Univariate Cox regression analysis of demographic and clinical factors.

Variable	Total (N)	Deaths (N)	HR (95% CI)	*p*-Value
**Age**	60	28	0.99 (0.96–1.02)	0.704
**Obesity**				
Yes	15	9	-	
No	45	19	0.58 (0.26–1.28)	0.178
**CV disease**				
Yes	11	2	-	
No	49	22	0.88 (0.36–2.17)	0.782
**HTA**				
Yes	40	18	-	
No	20	10	1.19 (0.55–2.57)	0.664
**LS**				
Yes	6	3	-	
No	54	25	0.73 (0.22–2.44)	0.615
**Phimosis**				
Yes	12	5	-	
No	48	23	1.32 (0.50–3.51)	0.574
**Smoking history**				
Yes	6	3	-	
No	54	25	1.18 (0.36–3.93)	0.784
**SES (rural)**				
Yes	33	15	-	
No	27	13	1.03 (0.49–2.17)	0.944

HR, hazard ratio; CI, confidence interval; CV, cardiovascular disease; SES, socio-economic status. Age was analyzed as a continuous variable (per year increase). *p*-values < 0.05 were considered statistically significant.

**Table 5:** Univariate Cox regression analysis of pathological and treatment-related factors.

Variable	Total (N)	Deaths (N)	HR (95% CI)	*p*-Value
**T-stage**				
T1	18	3	-	
T2	21	9	2.68 (0.72–9.96)	0.142
T3–T4	21	16	8.90 (2.57–30.8)	<0.001
**Tumor grade**				
G1	11	1	-	
G2	29	17	8.04 (1.07–60.6)	0.043
G3	20	10	7.52 (0.96–58.8)	0.054
**LVI**				
Yes	23	16	-	
No	37	12	0.25 (0.12–0.55)	<0.001
**PNI**				
Yes	10	7	-	
No	50	21	0.36 (0.15–0.86)	0.022
**Surgical margins**				
Negative (R0)	55	24	-	
Positive (R1)	5	4	3.30 (1.13–9.67)	0.030

HR, hazard ratio; CI, confidence interval; LVI, lymphovascular invasion; PNI, perineural invasion. *p*-values < 0.05 are considered statistically significant.

**Table 6:** Univariate Cox regression analysis of nodal stage and timing of second-stage surgery.

Variable	Total (N)	Deaths (N)	HR (95% CI)	*p*-Value
**N-stage**				
N0–1	29	7	-	
N2	21	14	4.07 (1.64–10.1)	0.003
N3	10	7	4.78 (1.66–13.8)	0.004
**LND timing**				
C1	11	1	-	
C2	24	15	9.93 (1.31–75.6)	0.027
C3	7	3	5.05 (0.53–48.6)	0.161
C4	18	9	7.27 (0.92–57.5)	0.060

HR, hazard ratio; CI, confidence interval; LND, lymph node dissection. C1, no indication for LND; C2, LND performed early; C3, delayed LND; C4, LND indicated but not performed. *p*-values < 0.05 are considered statistically significant.

**Table 8:** Final multivariate Cox regression model with independent prognostic factors.

Variable	Total (N)	Deaths (N)	HR (95% CI)	*p*-Value	GVIF	Adjusted GVIF
**Urethral invasion**					1.1	1.1
Yes	9	6	-			
No	51	22	0.32 (0.12–0.88)	0.027		
**T-stage**					1.6	1.1
T1	18	3	-			
T2	21	9	2.20 (0.56–8.69)	0.262		
T3–T4	21	16	8.26 (1.91–35.8)	0.005		
**N-stage**					1.7	1.1
N0–1	29	7	-			
N2	21	14	1.24 (0.40–3.86)	0.716		
N3	10	7	3.53 (1.13–11.00)	0.030		

HR, hazard ratio; CI, confidence interval; GVIF, generalized variance inflation factor. Final model selected by backward stepwise elimination based on *p*-values and collinearity diagnostics. Model C-index: 0.78 (95% CI: 0.69–0.87). *p*-values < 0.05 considered are statistically significant.

## Data Availability

The datasets generated and analyzed during the current study are available from the corresponding author on reasonable request, subject to institutional ethics committee approval and data protection regulations.

## Abbreviations

The following abbreviations are used in this manuscript:

PSCC	Penile squamous cell carcinoma
PC	Penile cancer
RT	Radiation therapy
CHT	Chemotherapy
ECE	Extracapsular extension
LVI	Lymphovascular invasion
PNI	Perineural invasion
LS	Lichen sclerosus
HPV	Human papillomavirus
QoL	Quality of life
OS	Overall survival
RMST	Restricted mean survival time
Cis	Confidence intervals
HR	Hazard ratio
VIFs	Variance inflation factors
BMI	Body mass index
TIP	Paclitaxel, cisplatin and ifosfamide
